# I kappa B kinase interacting protein as a promising biomarker in pan-cancer: A multi-omics analysis

**DOI:** 10.3389/fgene.2023.1138137

**Published:** 2023-03-14

**Authors:** Chenyang Bi, Zhe Wang, Yafei Xiao, Ying Zhao, Runjiang Guo, Luyao Xiong, Zhiyu Ji, Yifan Li, Quanying Li, Changjiang Qin

**Affiliations:** Department of Gastrointestinal Surgery, Huaihe Hospital of Henan University, Kaifeng, China

**Keywords:** IKBIP, pan-cancer, prognostic biomarker, methylation, immunosuppressive microenvironment

## Abstract

**Background:** Human chromosome 12 contains I kappa B kinase interacting protein (IKBIP) is also commonly known as IKIP. The involvement of IKBIP in the growth of tumors has only been discussed in a small number of publications.

**Purpose:** To explore the role that IKBIP plays in the development of a wide variety of neoplasms, as well as the tumor immunological microenvironment.

**Methods:** UALCAN, HPA, Genotype Tissue Expression, Cancer Genome Maps, and other datasets were used to analyze IKBIP expression. We thoroughly investigated the predictive importance of IKBIP in pan-cancer, clinical traits, and genetic anomalies. We studied whether there is a link between IKBIP and immune-related genes, microsatellite instability (MSI), and the incidence of tumor mutational burden (TMB). The link between immune cell infiltration and IKBIP expression was examined using data on immune cell infiltration from ImmuCellAI, TIMER2, and earlier studies. Finally, gene set enrichment analysis (GSEA) was performed to determine the signaling pathways associated with IKBIP.

**Results:** IKBIP is highly expressed in most cancers and is negatively associated with the prognosis of several major cancer types. Furthermore, IKBIP expression was linked to TMB in 13 cancers and MSI in seven cancers. Additionally, IKBIP is associated with numerous immunological and cancer-promoting pathways. Simultaneously, various cancer types have unique tumor-infiltrating immune cell profiles.

**Conclusion:** IKBIP has the potential to act as a pan-cancer oncogene and is crucial for both carcinogenesis and cancer immunity. Elevated IKBIP expression implies an immunosuppressive environment and may be used as a prognostic biomarker and therapeutic target.

## 1 Introduction

Cancer is caused by a series of complicated events that lead to uncontrolled cell growth and the ability of cells to mitigate natural cell death. This leads to malignancy and a high death rate (Sung et al., 2021). Despite significant advances in the treatment of a wide variety of tumors over the course of the last several years, the prognosis and survival rates for many types of cancer remain bleak (He et al., 2021). Thus, urgent action is required to identify novel and sensitive tumor biomarkers and additional therapeutic targets for the diagnosis and treatment of cancer (Liu et al., 2020).

I Kappa B Kinase Interacting Protein (IKBIP), also known as IKIP, is located on the human chromosome 12. This gene has received minimal attention from researchers. However, it was recently found that IKBIP is one of the target genes of p53, which is necessary for pro-apoptotic activity (Hofer-Warbinek et al., 2004). Recent findings have identified IKBIP as an essential inflammatory regulator (Wu et al., 2020). Additionally, IKBIP is critical for the development of glioma (Chen et al., 2020).

Genomic instability, epigenetic changes, oncogene activation, tumor suppressor gene suppression, and aberrant cell signaling (which results in the generation of abnormal proteins and stress signals) are all components of the multi-step, multi-layered process known as oncogenesis (Jeggo et al., 2016; Chatterjee et al., 2018). A key factor in the development of the malignant stage of cancer is disruption of the tumor microenvironment (TME), particularly the tumor immune microenvironment (TIME) (Shrihari, 2021). Given the extensive potential of the TIME in cancer treatment, it is essential to investigate the potential mechanism of tumors and identify new important indicators for tumor patients. Therefore, it is crucial to study the underlying mechanisms of tumors and identify novel crucial markers for cancer patients (Wu et al., 2022).

Here, we combined multiple databases [including The Cancer Genome Atlas (TCGA), Tumor Immune Estimation Resource (TIMER), UALCAN, Clinical Proteomics Cancer Analysis Consortium (CPTAC), and CBIopportAL databases] to systematically investigate the predictive significance of IKBIP in pan-cancer datasets. We looked at possible relationships between the expression of IKBIP and various immune-related genes, immune infiltration levels, tumor mutational burden (TMB), microsatellite instability (MSI), and the TME. Additionally, we used the Kyoto Encyclopedia of Genes and Genomes (KEGG) and Gene Set Enrichment Analysis (GSEA) in our investigation of the biological processes and pathways associated with IKBIP. Our research shows that IKBIP is associated with the emergence of numerous malignancies, holds promise as a novel immune checkpoint inhibitor, and has the potential to be a useful diagnostic, therapeutic, and prognostic marker. This research provides a foundation for understanding IKBIP’s mode of action in diverse malignancies and explains why immunotherapy treatments should target IKBIP.

## 2 Methods

### 2.1 Data collection

The TIMER database (https://cistrome.shinyapps.io/timer/) was used to examine the IKBIP expression profile and the prevalence of immune infiltrates in pan-cancer. Log2 TPM measurements were used to represent the gene expression levels. TCGA is a public platform with oncogene data. Using the UCSC Xena online database (https://xenabrowser.net/), the expression data for 33 tumor types, TMB data, MSI data, and clinical data were obtained (Goldman et al., 2020). The protein expression patterns in tumor and control tissues were also compared using UALCAN (http://ualcan.path.uab.edu/index.html), a database containing proteomics information generated from the CPTAC database. Methylation modification and genetic changes in tumor tissues were assessed using the cBioPortal website (https://www.cbioportal.org/) (Cerami et al., 2012).

### 2.2 Analyzing IKBIP expression and cancer clinicopathological characteristics or patient survival

Survival data were collected for each TCGA sample. Subsequently, we analyzed several markers to determine if IKBIP expression was associated with patient prognosis for developing various malignancies, such as overall survival (OS). We used the R packages “survminer” and “survival” to conduct Kaplan-Meier and log-rank test survival analyses across 33 cancer types (*p* < 0.05). Cox analysis with the Kaplan-Meier “survival” and “forestplot” R packages was used to analyze the correlation between IKBIP and survival. The use of the “ggpubr” and “limma” R packages allowed clinicopathological correlation analyses.

### 2.3 Immunohistochemistry staining of IKBIP

The Human Proteome Atlas (HPA) database (https://www.proteinatlas.org/) provides details on the distribution of proteins in human tissues and cells. We downloaded immunohistochemistry images of 10 distinct types of tumor tissues together with the equivalent normal tissues from the HPA to examine differential expression of IKBIP at the protein level. These included lung, stomach, pancreatic, cervical, endometrial, thyroid, liver, testicular, and thyroid cancers.

### 2.4 Analysis of the IKBIP diagnosis value

The clinical trait, tumor stage, was selected from each sample provided by TCGA; its relationship with IKBIP expression was examined using the “ggplot2” R tool. A type of drawing program called “ggplot2” can distinguish between drawing and data, drawing linked to data, and drawing unrelated to data. Using the “pROC” tool, the diagnostic accuracy of IKBIP was determined by ROC curve analysis based on sensitivity and specificity. AUC values may range from 1 (good diagnosis) to 0.5 (no diagnostic value) (Smoot et al., 2011).

### 2.5 Multiple cancer types show an association between IKBIP expression and TMB, and IKBIP expression and MSI

Thirty-three tumors were evaluated using TMB with Perl scripts to count somatic mutations, which were then rectified by dividing with the exon length. TCGA was used to extract the MSI scores. The “cor.test” command, which is based on Spearman’s approach, was used to examine the correlation between IKBIP expression and either TMB or MSI. Radar plots were generated using the “fmsb” R package.

### 2.6 IKBIP expression is correlated with tumor cell infiltration and immune modulator genes in a pan-cancer analysis

Using the Genomic Data Commons (GDC) data gateway website, information for 33 distinct cancer types and healthy tissues was downloaded from the TCGA. Immuneeconv was used. It is an R software package that combines the two most recent algorithms, TIMER and xCell, to accurately evaluate immune scores. Spearman correlation analysis was used to construct a heat map depicting the immunological score, genes related to immune checkpoints, and IKBIP gene expression in various cancers. In the heat maps, the horizontal axis depicts various cancer types, and the vertical axis depicts various immunological scores, colors, and correlation coefficients. Statistical analysis was performed using R software (version 4.2.1; ∗*p* < 0.05, ∗∗*p* < 0.01, ∗∗∗*p* < 0.001). The R packages “ggplot2,” “ggpubr,” and “ggExtra” were then used to examine the relationship between IKBIP and TME infiltration (with a cutoff value of *p* < 0.001).

### 2.7 Correlation of IKBIP expression with DNA methylation

The methylation status of IKBIP in different cancers and related tissues was examined using the UALCAN database (http://ualcan.html). Student’s t-test was used to determine the statistical significance of differences. Differences were considered statistically significant at *p* < 0.05.

### 2.8 Prediction of target miRNAs using IKBIP and construction of the ceRNA network

IKBIP target miRNAs were retrieved from five miRNA prediction databases: DIANA-microT (http://diana.imis.athena-innovation.gr/DianaTools/index.php?r=microT_CDS/index), miRDB (http://mirdb.org/miRDB/), miRWalk (http://mirwalk.umm.uni-heidelberg.de/), and TargetScan StarBase (https://www.targetscan). The intersection of the miRNAs predicted by the five databases is known as the target miRNA. To provide lncRNA and circRNA information regarding IKBIP, StarBase v2.0 was used to create the most thorough miRNA-lncRNA and miRNA-circRNA interaction networks. Mammal, human, hg19, and strict stringency 5) of CLIP-Data, and with or without data of Degradome-Data, were the screening requirements. Using Cytoscape, we were able to determine how ceRNAs collaborated with one another in terms of mRNA, miRNA, and ncRNA interactions.

### 2.9 IKBIP is expressed alongside immune-related genes and pathways in malignancies

For co-expression studies, the R packages “RColorBrewer,” “limma,” and “reshape2” were utilized. GSEA (https://www.gseamsigdb.org/gsea/downloads.jsp) was used to gather gene sets from Gene Ontology (GO) and KEGG databases. R packages called “limma,” “org. Hs. eg.db,” “clusterProfiler,” and “enrichplot” were used to investigate enriched pathways, as well as GO and KEGG functional annotations, in relation to IKBIP.

### 2.10 Analytical statistics

The non-parametric Mann-Whitney test was used to compare the transcriptome expression patterns of IKBIP in cancer and healthy control tissues according to the TIMER database. The protein expression pattern and methylation level of IKBIP in tumor tissues and normal control tissues were analyzed using Student’s *t*-test based on the UALCAN database. Patients were categorized according to their IKBIP expression levels, and Kaplan-Meier analysis was used to assess their overall survival. All statistical tests were performed at a significance level of *p* < 0.05.

## 3 Results

### 3.1 Pan-cancer expression landscape of IKBIP

**FIGURE 1:**
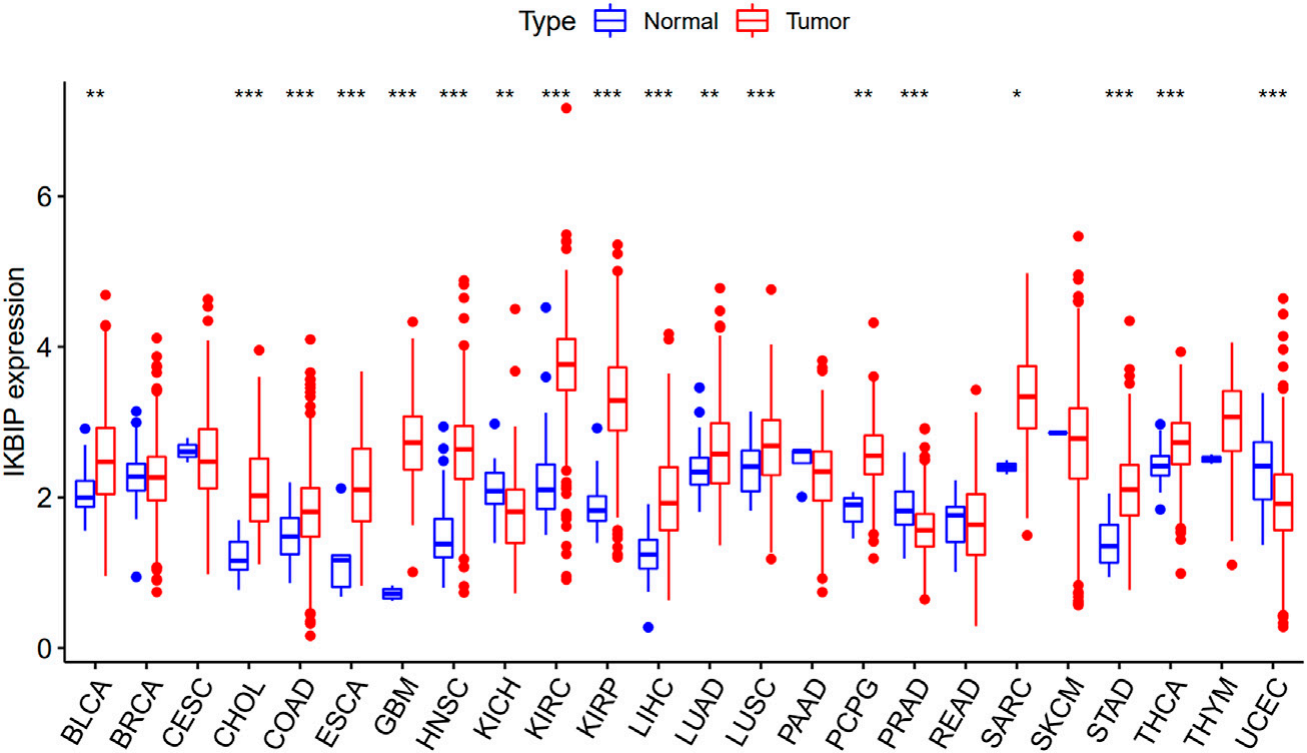
Upregulated mRNA expression of IKBIP in pan-cancer. IKBIP expression levels across cancer types, as reported by the TCGA. (*p < 0.05, **p < 0.01, ***p < 0.001).

We directly compared IKBIP expression using TCGA data. Increased IKBIP mRNA expression in tumor tissues was consistently observed compared to normal tissues in patients with 33 malignancies (*p* < 0.05) (including BLCA, CHOL, COAD, ESCA, GBM, HNSC, KIRC, KIRP, LIHC, LUAD, LUSC, PCPG, SARC, STAD, and THCA) (Figure 1).

**FIGURE 2:**
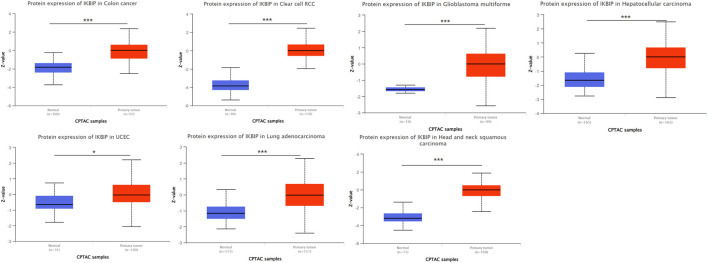
Determine the relative abundance of IKBIP protein in the indicated tumor types using the Ualcan database. *p < 0.05, **p < 0.01, ***p < 0.001.

Further analysis of protein level was performed in the CPTAC and HPA databases. The CPTAC database provides only seven tissues available for analysis. Among them, IKBIP was highly expressed in COAD, KIRC, LUAD, HNSC, GBM and HNSC, which was consistent with the results of mRNA level. However, it is noteworthy that it is lowly expressed in UCEC, which is in contrast to the mRNA level (Figure 2). In addition, we found another four tissue expressions from the HPA database for protein level validation. Among them, IKBIP was highly expressed in THCA, BLCA, and STAD tissues, which was consistent with the results at mRNA level. IKBIP was lowly expressed in PRAD tissues, which was opposite to mRNA level (Supplementary Figure S1).

We also evaluated the diagnosis capacity of IKBIP and constructed ROC curves to examine its ability to predict the diagnosis of patients with different malignancies. The data revealed that AUC >0.7 was only present in nine malignancies, and IKBIP had the highest diagnosis value for GBM (AUC = 0.997, CI: 0.994–1), and KIRC (AUC = 0.957, CI: 0.929–0.985) (Supplementary Figure S2). In conclusion, IKBIP is differentially expressed and highly sensitive and specific in some cancers, suggesting that IKBIP may play a potentially critical role in cancer diagnosis.

### 3.2 Pan-cancer IKBIP genetic alteration

**FIGURE 3:**
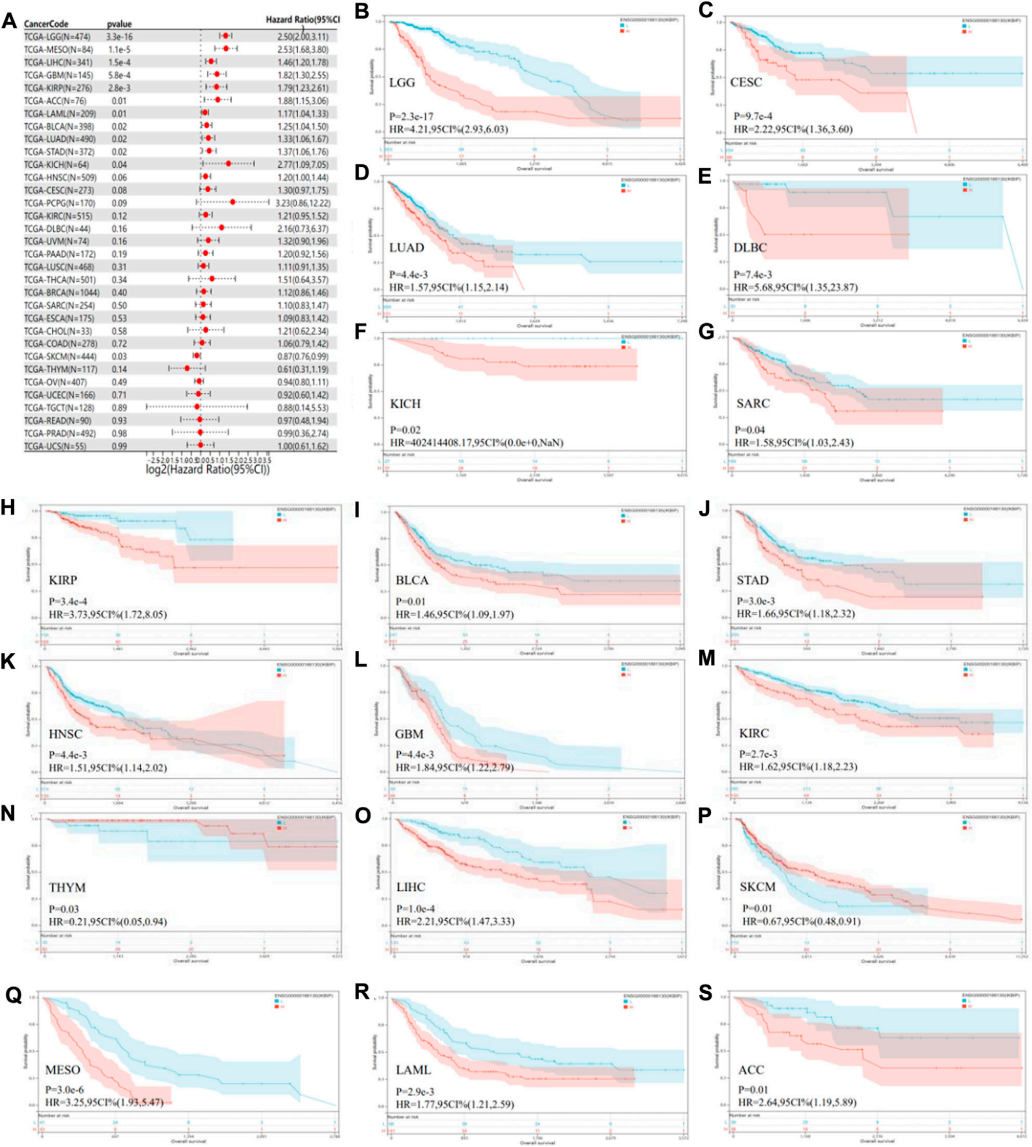
Pan-cancer OS study of IKBIP. **(A)** IKBIP pan-cancer hazard ratios are displayed on a forest plot. **(B–S)** TCGA database analysis of pan-cancer survival curves with high and low IKBIP expression.

By analyzing the correlation between IKBIP expression and prognosis, we found that OS was significantly lower in patients with IKBIP overexpression compared to those with IKBIP non-expression in ACC (*p* = 0.01), BLCA (*p* = 0.02), GBM (*p* = 5.8e-4), KICH (*p* = 0.04), KIRP (*p* = 2.8e-3), LAML (*p* = 0.01), LGG (*p* = 3.3e-16), LIHC (*p* = 1.5e-4), LUAD (*p* = 0.02), MESO (*p* = 1.1e-5), STAD (*p* = 0.02), and SKCM (*p* = 0.03) (Figure 3). According to our findings, IKBIP expression was a significant risk factor for ACC, BLCA, GBM, KICH, KIRP, LAML, LGG, LIHC, LUAD, MESO, and STAD, particularly KICH (hazard ratio = 2.77). In contrast, IKBIP expression in SKCM was a sign of low risk. In addition, Kaplan-Meier survival analysis revealed that high IKBIP expression was associated with higher OS in THYM and SKCM patients. However, IKBIP expression was associated with lower OS in patients with LGG, CESC, LUAD, DLBC, KICH, SARC, KIRP, BLCA, STAD, HNSC, GBM, KIRC, LIHC, MESO, LAML, and ACC.

**FIGURE 4:**
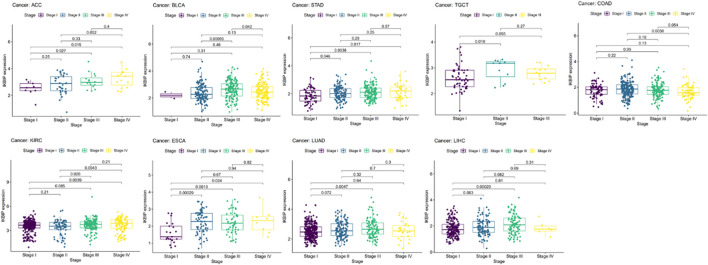
IKBIP’s prognostic significance in many malignancies. Cancer stage and IKBIP transcript levels are correlated.

To further elucidate IKBIP’s therapeutic benefits in various malignancies, the relationship between the level of IKBIP transcription and stage of malignancy was examined. ACC, BLCA, STAD, TGCT, COAD, KIRC, ESCA, LUAD, and LIHC cancer stages were strongly associated with IKBIP mRNA expression (*p* < 0.05) (Figure 4). IKBIP transcription varies significantly among various cancer stages in these malignancies.

IKBIP is associated with patient staging and prognosis in some cancer types, suggesting that IKBIP may have the potential to be a prognostic marker.

### 3.3 Correlation analysis of IKBIP expression with tumor mutational load, microsatellite stability, immune checkpoint genes, and tumor microenvironment

**FIGURE 5:**
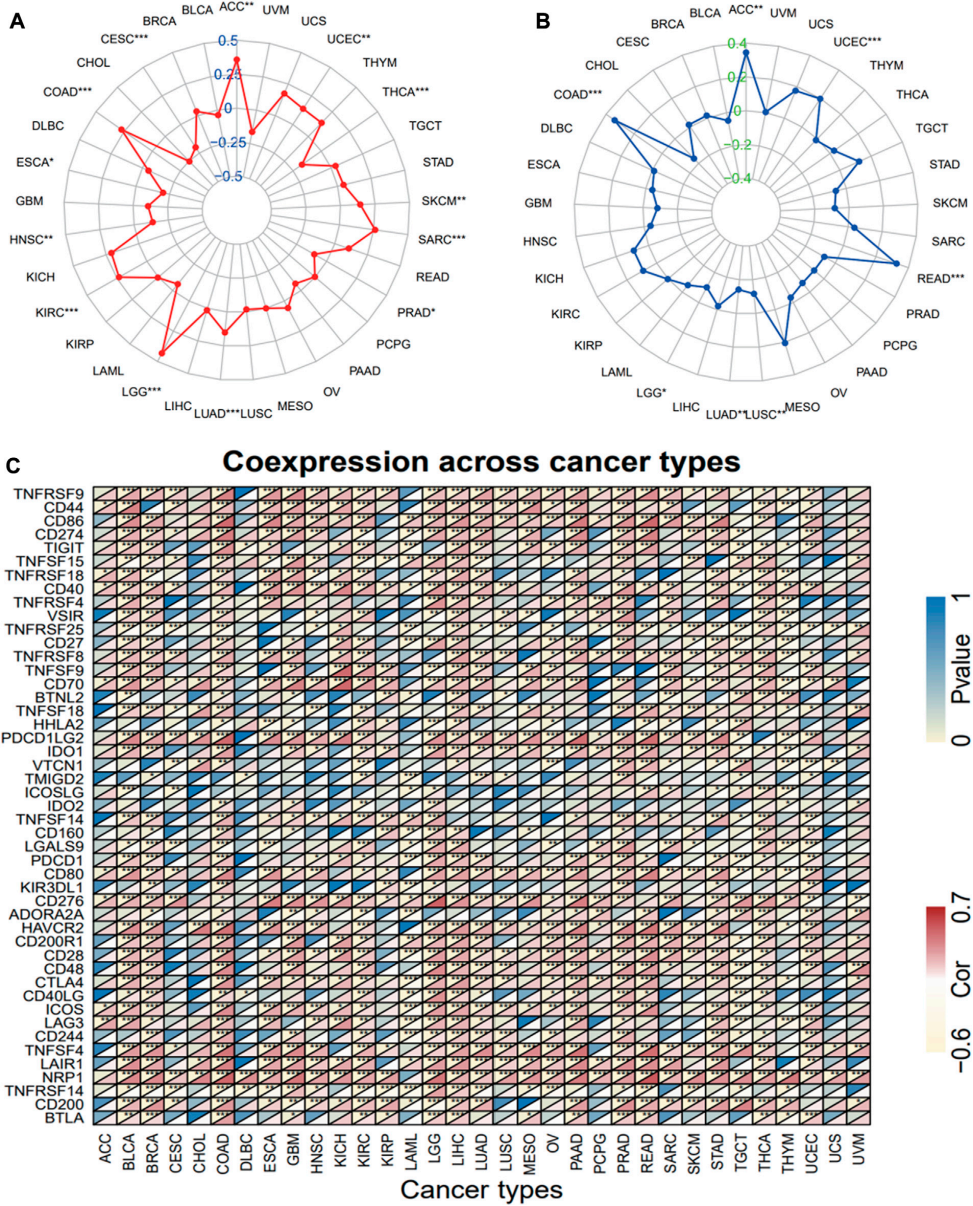
Correlation between TMB, MSI, and ICGs and IKBIP expression. **(A)** Relationship between TMB and IKBIP expression. **(B)** MSI and IKBIP expression are correlated. **(C)** Relationship between ICGs and IKBIP expression.

We examined the relationship between IKBIP expression and TMB, MSI, and ICGs to determine whether IKBIP could be used as a biomarker to measure the efficacy of immunotherapy. TMB has been shown to be directly linked to the efficacy of immunotherapy in a number of tumor types (Yarchoan et al., 2017). In 13 pan-cancer subtypes, IKBIP expression was linked to TMB levels (Figure 5A). In particular, TMB levels were favorably correlated with IKBIP expression levels in ACC, COAD, KIRC, LGG, LUAD, SARC, SKAM, and UCEC. In contrast, IKBIP expression was inversely associated with TMB levels in CESC, ESCA, HNSC, PRAD, and THCA.

Similarly, MSI is a known prognostic marker for immunotherapy activity (Dudley et al., 2016). Here, we examined the correlations between IKBIP expression and MSI in datasets related to all types of cancer (Figure 5B). Our findings showed that IKBIP expression was favorably correlated with MSI levels in ACC, COAD, READ, and UCEC, whereas it was negatively correlated with MSI levels in CHOL, LGG, LUAD, and LUSC. ICGs are a practical measure of the effectiveness of immunotherapy (Larkin et al., 2015); hence, we conducted a correlation analysis between the expression of ICGs and IKBIP in different pan-cancer subtypes (Figure 5C). Notably, IKBIP expression was positively correlated with the majority of ICGs in the CPAD, LGG, and LIHC groups, demonstrating a strong correlation with several ICGs. IKBIP expression in LAML and TGCT had a significant inverse relationship with the vast majority of ICGs, to look at things from a different perspective.

**FIGURE 6:**
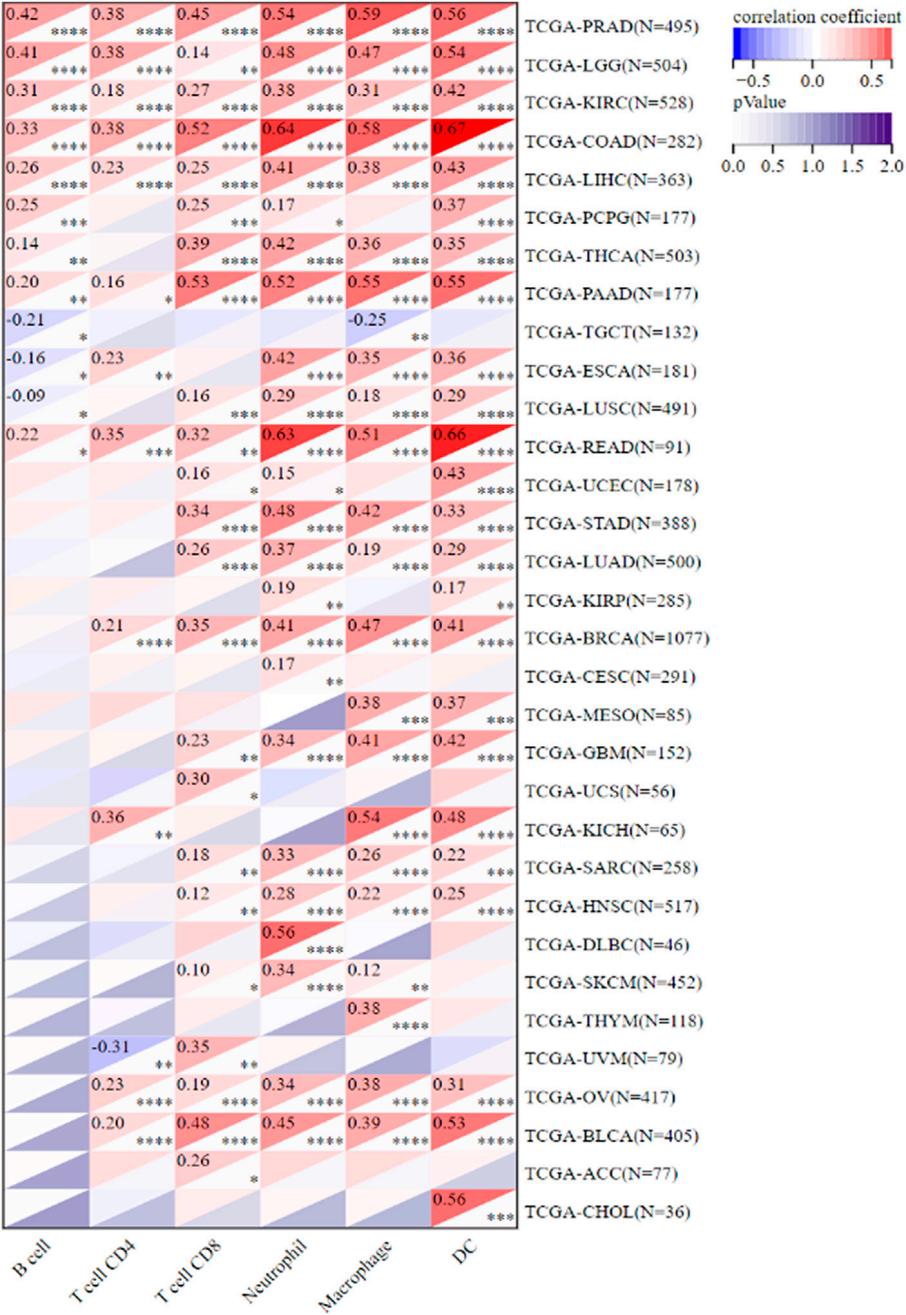
IKBIP expression was linked with immune infiltration. IKBIP expression had a high connection with immune cell infiltration levels in the TIMER database.

Because of the one-of-a-kind dynamics that exist between IKBIP and the immune response, we used the TIMER database to carry out an in-depth investigation into the connection between the level of IKBIP expression and the extent to which immune cells are present in different types of cancer. Figure 6 demonstrates a substantial correlation between the expression of IKBIP and the number of invading immune cells (including B cells in 12 cancer types, CD4^+^ T cells in 13, CD8^+^ T cells in 23, macrophages in 23, neutrophils in 24, and DCs in 24 cancer types). The five types of immune pathways are chemokine, receptor, MHC, immuno-inhibitory, and immunostimulatory pathways. These findings demonstrated that IKBIP gene expression was positively correlated with immunomodulatory genes in the majority of malignancies (Supplementary Figure S3).

**FIGURE 7:**
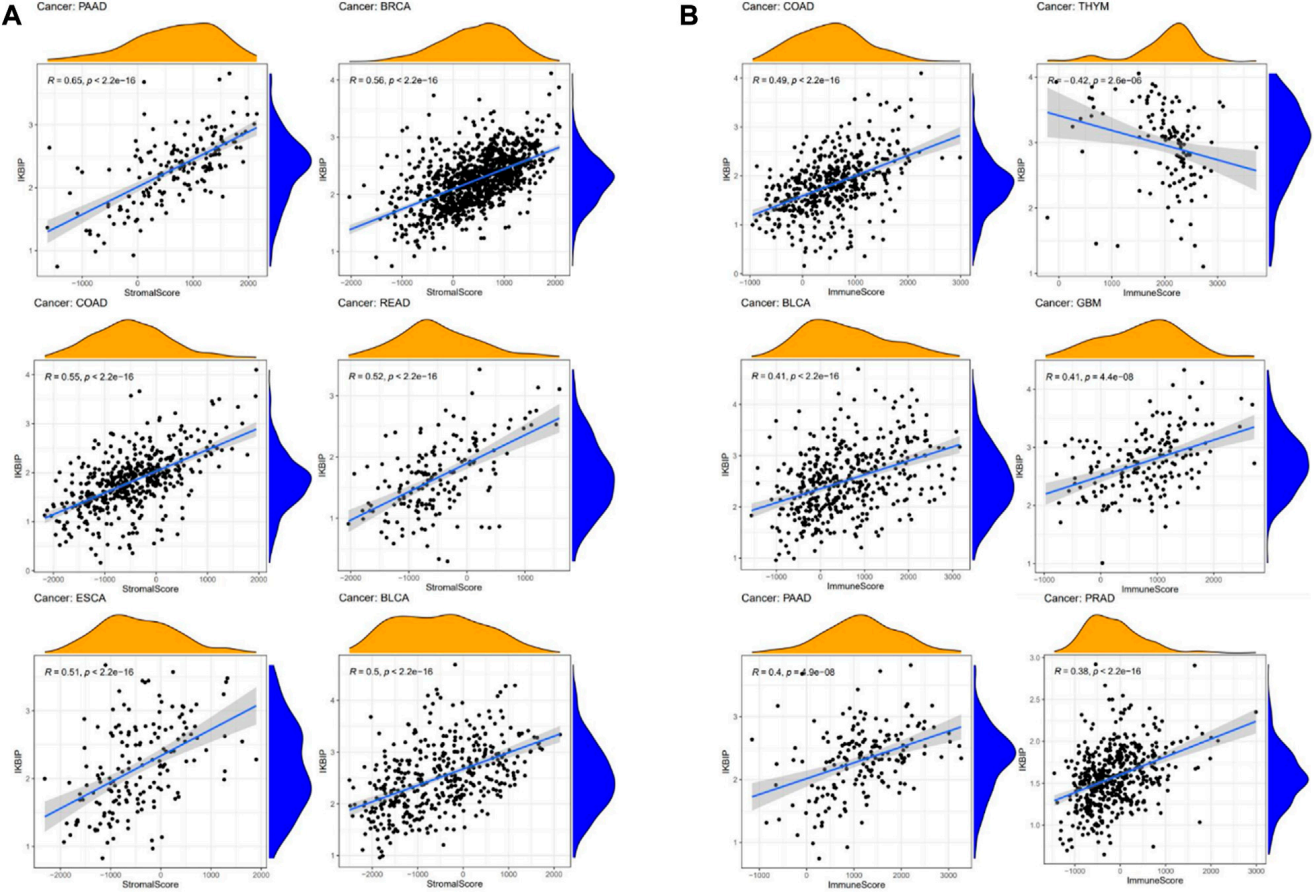
Six cancers with the highest association coefficients between IKBIP expression and the TME. **(A)** IKBIP expression was positively associated with stromal scores in pancreatic cancer (PAAD), breast cancer (BRCA), colon cancer (COAD), rectal cancer (READ), esophageal cancer (ECSA) and bladder urothelial carcinoma (BLCA). **(B)** IKBIP expression was negatively correlated with immune scores in thymic carcinoma (THYM) and positively correlated with immune scores in thyroid cancer colon cancer (COAD), kidney clear cell carcinoma (BLCA), glioblastoma multiforme (GBM), pancreatic cancer (PAAD), and prostatic cancer (PRAD).

We further used the xCell online tool to examine the relationship between IKBIP expression and infiltration of different types of immune cell subtypes. Among the immune cell subtypes, we found that IKBIP in COAD, LGG, BLCA, PRAD, STAD, BRCA and READ was negatively correlated with these immune cell subtypes and positively correlated in THYM, OV, LAML tissues. In addition, IKBIP expression correlated most strongly with Th2 cells and CLP cells in various malignancies (Supplementary Figure S4). Using the ESTIMATE approach, the stromal and immunological scores of 33 tumors were analyzed, and their connections with IKBIP expression were investigated. Figure 10 lists the six cancer types with the highest correlation coefficients between the TME and IKBIP expression. The results showed that IKBIP expression was positively correlated with stromal scores in PAAD, BRCA, COAD, READ, ESCA, and BLCA (Figure 7A). Additionally, IKBIP expression was positively correlated with immune scores in COAD, BLCA, GBM, PAAD, and PRAD, and significantly adversely correlated with immune scores in THYM (Figure 7B).

Overall, these results suggest that IKBIP expression is correlated with TMB, MSI, ICGs, and TME in multiple pan-cancer datasets, convincingly indicating that it could be a robust and reliable biomarker for predicting the responses of cancer cells to immunotherapy.

### 3.4 Pan-cancer analysis of the methylation level and genetic alteration of IKBIP

**FIGURE 8:**
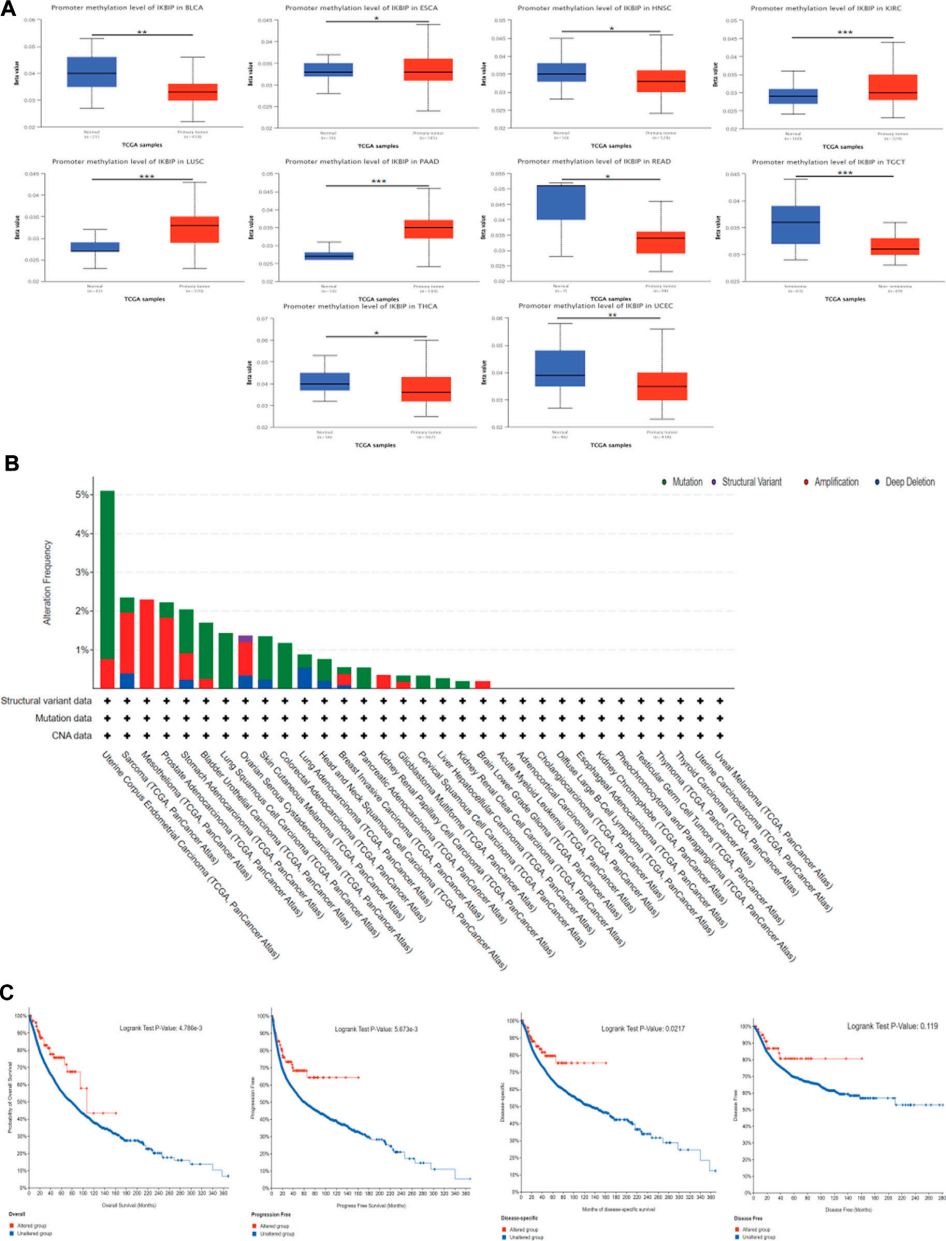
DNA methylation and mutation features of IKBIP in pan-cancer. **(A)** IKBIP promoter methylation in pan-cancer. **(B)** Using the cBioPortal database, the modification frequency with various mutation types was studied. **(C)** Utilizing the cBioPortal database, we analyzed how IKBIP mutation status affected overall, disease-specific, disease-free, and progression-free survival in cancer patients. ∗*p* < 0.05, ∗∗*p* < 0.01, and ∗∗∗*p* < 0.001).

We utilized the UALCAN and TCGA databases to examine IKBIP DNA methylation. According to the UALCAN database, BLCA, ESCA, HNSC, READ, TGCT, THCA, and UCEC tissues had significantly lower IKBIP methylation levels than normal tissues (Figure 8A). On the other hand, elevated levels of IKBIP methylation have been found in KIRC, LUSC, and PAAD. This suggests that methylation is an important way for IKBIP to exert its biological effects, and that methylation patterns differ in different tumor tissues, including hypomethylation and hypermethylation.

We also investigated pan-cancer alterations in IKBIP using the cBioPortal (TCGA Pan-Cancer Atlas) database. The findings showed that patients with mesothelioma tumors had an IKBIP change frequency of up to 2.3% (Figure 8B since amplification is the most common genetic modification, among all others. We also investigated the latent association between genetic changes in IKBIP and patient prognosis for various cancer types. As shown in Figure 8C, patients with tumors and genetic changes in IKBIP had better OS, progression-free survival (PFS), and disease-specific survival (DSS) than patients without changes. However, DFS did not differ between the groups.

### 3.5 Construction of a co-expression network and prediction of target ncRNAs

**FIGURE 9:**
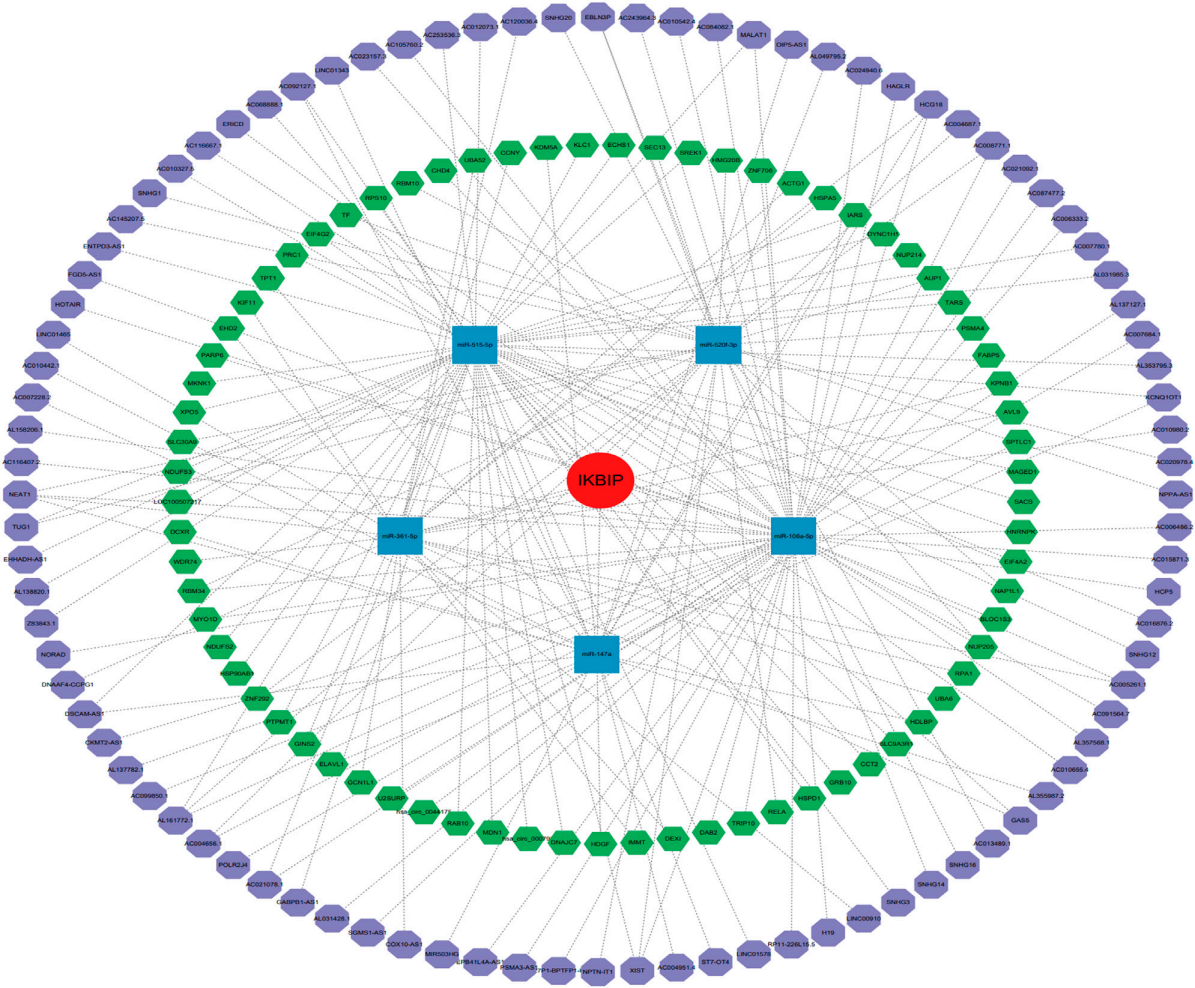
IKBIP’s ceRNA network. IKBIP ceRNA networks (red circle represents the hub gene, blue squares represent the miRNAs, green hexagons represent lncRNAs, and purple hexagons represent circRNAs).

It is generally known that miRNAs can combine with mRNAs to suppress gene expression and cause gene silencing. The relationship between mRNAs, miRNAs, and associated non-coding RNAs is based on the interaction of the competing endogenous RNAs network. ncRNAs, such as circular RNAs (circRNAs) and long non-coding RNA (lncRNAs), are thought to be upstream molecules that can affect the function of miRNAs by interacting with miRNA response regions, thereby increasing gene expression (Salmena et al., 2011). Therefore, we investigated the ceRNA networks that may regulate IKBIP expression in various tumors. First, we screened the miRNAs of IKBIP in miRWalk, miRDB, TargetScan, DIANA-microT, and StarBase v2.0, and used their intersection to obtain five miRNAs (namely, hsa-miR-520f-3p, hsa-miR-515-5p, hsa-miR-361-5p, hsa-miR-147a, and hsa-miR-106a-5p) (Supplementary Figure S5). Subsequently, the miRNA that was obtained was used to predict its circRNA and lncRNA using the StarBase database, and the lncRNA that was obtained was used to predict its circRNA using the StarBase database. Consequently, 92 and 73 target circRNAs and lncRNAs, respectively, were identified. The prediction results were in accordance with the ceRNA networks depicted in Figure 9, which may serve as a foundation for our investigation of possible medicines that modulate IKBIP.

### 3.6 Drug sensitivity analysis of IKBIP

As the role of drug resistance in cancer has been gaining attention, we further investigated the analysis of potential correlations between drug sensitivity and IKBIP expression using the CellMiner™ database. Our results showed that the expression of IKBIP was positively correlated with simvastatin, P-529, sulforaphane, teratinib, and midostatin. The expression of IKBIP was negatively correlated with DOLASTATIN 10, BMS-387032, Tamoxifen, EMD-534085, Vinorelbine, TYROTHRICIN, Barasertib, Homoharringtonine, ARQ-621, Paclitaxel SR16157, PF-2771 and ARRY-520 Isomer A (Supplementary Figure S6). Notably, IKBIP expression had the strongest positive correlation with Sarivastatin, and IKBIP expression had the highest negative correlation with DOLASTATIN 10.

### 3.7 Enrichment analysis of cancers in different groups

**FIGURE 10:**
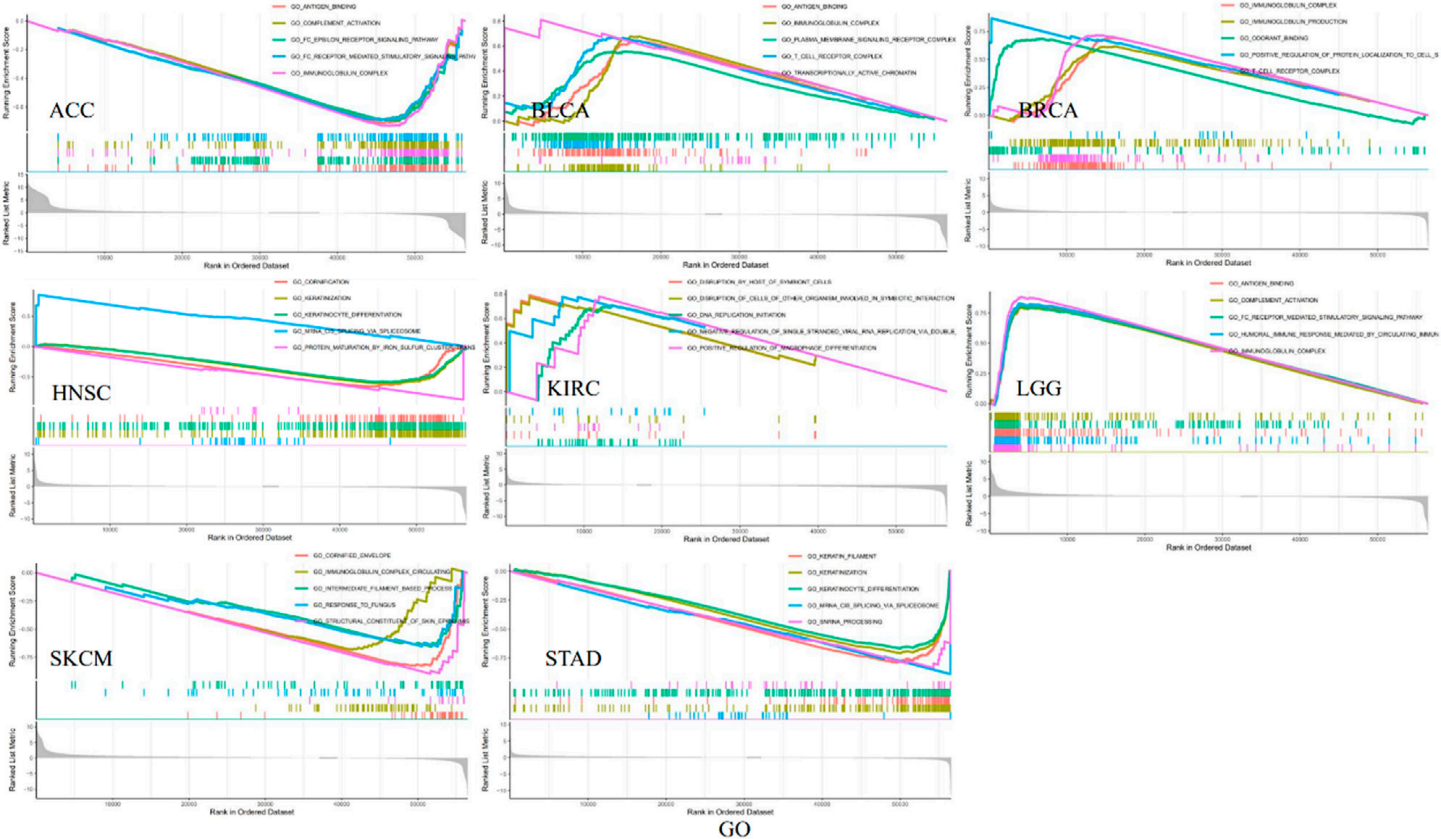
GO functional terms of IKBIP in various each cancer type in the high-risk group and low-risk group.

GO functional annotations and KEGG pathways related to IKBIP in different malignancies were examined (Figure 10). According to the results, IKBIP was linked to the negative regulation of immune-related activities in the ACC, including antigen binding, complement activation, FC epsilon receptor signaling pathway, and immunoglobulin complex (Figure 10). The negative modulation of immune-related activities by the circulating immunoglobulin complex was associated with IKBIP in SKCM (Figure 10).

Several tumorigeneses and immunological pathways, such as “ECM-receptor interaction,” “NOD similar receptor signaling route,” “Chemokine signaling pathway,” and “Cytokine receptor interaction,” were found to be under the potential control of IKBIP in a KEGG pathway analysis (Supplementary Figure S7).

## 4 Discussion

The incidence and mortality of cancer have been rising significantly globally (Siegel et al., 2019). Even the most common cancer therapies, such as chemotherapy, radiation therapy, and surgery to remove malignant tissue, are not particularly effective (Abril-Rodriguez and Ribas, 2017). Immune checkpoint blockade therapy, which has emerged as one of the most promising immunotherapies for cancer treatment in recent years, has fundamentally altered the approach used to treat cancer (Palmieri and Carlino, 2018). A block in the immune system is removed by immune checkpoint blocking medication, which also prompts a long-lasting anticancer response (Schaub et al., 2018; Deng et al., 2020; Sheng et al., 2020; Xu et al., 2020). Pan-cancer analysis can highlight tumor similarities and distinctions, shedding light on how to avoid cancer and create treatment targets (ICGC/TCGA Pan-Cancer Analysis of Whole Genomes Consortium, 2020). Many recent studies have focused on pan-cancer analysis of the entire genome, finding mutations, RNA changes, and driver genes that are associated with the occurrence and progression of cancer. This is significant for early cancer detection and biomarker creation (Bailey et al., 2018; Liu et al., 2019; Calabrese et al., 2020; Rheinbay et al., 2020; Rodriguez-Martin et al., 2020).

There are only a few articles on IKBIP as a predictive/prognostic biomarker, and all of them are about gliomas. Yang et al. (2021). studied the transcriptional expression profile of IKBIP in 998 glioma patients and found that IKBIP expression was significantly and positively correlated with the World Health Organization (WHO) glioma grade. They concluded that IKBIP is a novel EMT-related biomarker that predicts poor survival in gliomas. Liang et al. (2021) suggested that circ_0072391 aggravates glioma through miR-338-5p/IKBIP axis. Chen et al. (2020) identified IKBIP as a novel biomarker that may be relevant to the diagnosis, treatment and prognosis of glioma. In addition, a recent article identified IKBIP as a novel glioblastoma biomarker that maintains abnormal tumor cell proliferation by inhibiting the ubiquitination and degradation of CDK4 (Li et al., 2023). Overall, their findings are consistent that IKBIP is highly expressed in gliomas and is associated with significantly shorter patient survival.

In our study, we first examined the expression of IKBIP. IKBIP mRNA was strongly expressed in BLCA, CHOL, COAD, ESCA, GBM, HNSC, KIRC, KIRP, LIHC, LUAD, LUSC, PCPG, SARC, STAD, and THCA tissues compared to normal tissues. On the other hand, low expression was observed in KICH, PRAD and UCEC. In COAD, GBM, HNSC, KIRC, LIHC, THCA, BLCA, STAD, UCEC, and LUAD, mRNA levels were consistent with protein expression levels. However, high expression was observed in PRAD and UCEC in contrast to their mRNA levels. Kaplan-Meier OS analysis demonstrated that higher IKBIP expression was associated with worse OS in several cancers, including LGG, CESC, LUAD, DLBC, KICH, SARC, KIRP, BLCA, STAD, HNSC, GBM, KIRC, LIHC, MESO, LAML, and ACC. In several cancer types, IKBIP expression has been linked to immune invasion and immunological checkpoint markers. According to our GSEA analysis, IKBIP was strongly linked to a number of signaling pathways, such as the fc-epsilon-receptor-signaling pathway, fc-receptor-mediated-stimulatory-signaling pathway, and keg-nod-like-receptor-signaling pathway. These findings suggested that this gene plays an important role in cancer development.

Previous research has demonstrated that TMB represents the total neoantigen load, which affects the effectiveness of immunotherapy (Wu and Dai, 2017; Cristescu et al., 2018). TMB may also be helpful as a potential pan-cancer prognostic biomarker, offering a direction for choosing immunotherapy in the era of precision medicine (Samstein et al., 2019). The study also discovered that TMB was related to clinical ICI response, with a higher TMB corresponding to a higher OS rate (da Silva et al., 2021). MSI is another important biological indicator of ICI responsiveness. This study showed an association between IKBIP expression and TMB in 13 malignancies (including ACC, UCEC, THCA, SKCM, SARC, PRAD, LUAD, LGG, KIRC, HNSC, ESCA, COAD, and CESC), and with MSI in seven malignancies (namely, ACC, UCEC, READ, LUSC, LUAD, LGG, and COAD). These results suggest that TMB and MSI of different cancers are influenced by IKBIP expression, which in turn influences the patient’s response to ICI therapy. Thus, the prognosis and responsiveness of diverse forms of cancer to immunotherapy should be determined. We hypothesized that high IKBIP expression and high TMB and MSI expression predict improved prognosis and responsiveness to ICI treatment in tumor types where IKBIP expression is positively linked with TMB. This hypothesis is based on both prior research and our findings.

Crucial to tumor growth and metastasis is the TME (Binnewies et al., 2018). An increasing amount of data points to the clinicopathological importance of TME in predicting the survival status and treatment outcomes of tumor patients (Zhang and Zhang, 2020). Although immunotherapy has made great strides in treating cancer, there are still many things that prevent it from being widely used (Murciano-Goroff et al., 2020; Wang et al., 2021). Consequently, the discovery of new targets and biomarkers is key for significantly boosting immunotherapy efficacy. Thus, it is important to know the immune infiltration status of cancer patients to choose the best personalized immunotherapy plan (Murciano-Goroff et al., 2020; Wang et al., 2021). The function of IKBIP and its effects on the tumor immune microenvironment have not been fully investigated. This study examined the immunological status of patients with cancer and discovered a connection between IKBIP and tumor immune cells by measuring IKBIP expression. IKBIP has a significant relationship with the infiltration of B cells, CD8^+^ T cells, CD4^+^ T cells, macrophages, neutrophils, and dendritic cells (DC) across a wide range of cancers. The relationship between IKBIP expression and immunosuppressive and immunostimulatory genes was also investigated. We used TCGA data for pan-cancer transcriptome analysis and found that IKBIP expression was positively correlated with the matrix scores of PAAD, BRCA, COAD, READ, ESCA, and BLCA. In addition, IKBIP expression was significantly negatively correlated with the immune score of THYM and positively correlated with the immune scores of COAD, BLCA, GBM, PAAD, and PRAD. Our findings imply that IKBIP expression is strongly associated with tumor immune infiltration, which influences patient prognosis and offers a potential immunotherapeutic target for the treatment of patients with various cancers.

DNA methylation is one of the most common epigenetic modifications, a biological process that adds methyl groups to DNA molecules. Methylation can alter the activity of DNA fragments without changing the sequence (Jian et al., 2023). DNA methylation usually alters the structure, stability and shape of chromatin to prevent gene expression (Wang et al., 2021). Aberrant DNA methylation disrupts transcriptional regulation, predisposes cells to malignant transformation, and is a hallmark of human cancer (Kulis and Esteller, 2010). Hypomethylation is usually associated with chromosomal instability and imprinting loss, whereas hypermethylation is associated with promoters and secondary to gene (oncogene/suppressor gene) silencing (Gonzalo, 2010). Tumor suppressor genes are often silenced or turned off when cancer cells have excessive methylation in the promoter region (Wang and Lei, 2018; Mehdi and Rabbani, 2021; Wang et al., 2021). DNA methylation provides a potential biomarker for the early diagnosis and prognosis of cancer (Hao et al., 2017; Kim et al., 2018; Li et al., 2018; Ma et al., 2020), but there is a lack of studies on IKBIP methylation. Our analysis showed that IKBIP was significantly different in DNA methylation in ten common malignancies, seven of which were hypermethylated and three of which were hypermethylated. In comparison with mRNA levels, most of them are consistent with previous researchers’ knowledge that there is hypermethylation of oncogenes and hypomethylation of oncogenes (Gonzalo, 2010), such as in BLCA, ESCA, HNSC, THCA; but there are also inconsistencies, such as in UCEC, KIRC, LUSC and this phenomenon needs further study in the future.

To the best of our knowledge, this work is the first comprehensive pan-cancer examination of IKBIP. This study rigorously examined the data on all types of cancer from several databases. However, our study had some limitations. First, in the earlier findings of the analysis of distinctly expressed genes, we did not find that IKBIP was differentially expressed between some cancer tissues (BRCA, CESC, PAAD, READ, SKCM, and THYM) and normal tissues. Therefore, additional case validation is required to confirm the diagnostic and prognostic utility of IKBIP for BRCA, CESC, PAAD, READ, SKCM, and THYM. Second, additional experimental research is required to confirm these bioinformatic findings because our study was restricted to the analysis of pre-existing data. The role of IKBIP at the molecular level will become clearer in future studies. Third, although the results of the pan-cancer analysis showed that IKBIP expression was linked to immune cell infiltration and immunomodulatory mechanisms, further research is needed to determine the underlying mechanism.

## 5 Conclusion

IKBIP was variably expressed in tumor and non-tumoral tissues, as well as during various stages of tumor development, according to our analysis of IKBIP expression across all cancer types. This study also demonstrated a relationship between IKBIP and clinical prognosis. According to our findings, IKBIP can be used as a standalone prognostic factor for a variety of malignancies. The various expression levels were linked to various clinical outcomes, which calls for more research into the precise function of IKBIP in each cancer type. IKBIP expression levels were associated with tumor immune invasion and IKBIP-targeted drugs, and IKBIP was found to be positively or negatively correlated with TMB and MSI in various malignancies. This could offer fresh perspectives on how to use IKBIP to diagnose and treat human pan-cancers.

## Data Availability

The original contributions presented in the study are included in the article/Supplementary Material, further inquiries can be directed to the corresponding author.

## Glossary

ACC: adrenocortical carcinoma
BLCA: bladder urothelial carcinoma
BRCA: breast invasive carcinoma;
CESC: cervical squamous cell carcinoma and endocervical adenocarcinoma
CHOL: cholangiocarcinoma
COAD: colon adenocarcinoma
DLBC: diffuse large B-cell lymphoma
ESCA: esophageal carcinoma
GBM: glioblastoma multiforme
HNSC: head and neck squamous cell carcinoma
KICH: kidney chromophobe
KIRC: kidney renal clear cell carcinoma
KIRP: kidney renal papillary cell carcinoma
LAML: acute myeloid leukemia
LGG: lower grade glioma
LIHC: liver hepatocellular carcinoma
LUAD: lung adenocarcinoma
LUSC: lung squamous cell carcinoma
MESO: mesothelioma
OV: ovarian serous cystadenocarcinoma;
PAAD: pancreatic adenocarcinoma
PCPG: pheochromocytoma and paraganglioma
PRAD: prostate adenocarcinoma
READ: rectum adenocarcinoma
SARC: sarcoma
SKCM: skin cutaneous melanoma
STAD: stomach adenocarcinoma
TGCT: testicular germ cell tumor
THCA: thyroid carcinoma
THYM: thymoma
UCEC: uterine corpus endometrial carcinoma
UCS: uterine carcinosarcoma
UVM: uveal melanoma
TCGA: The Cancer Genome Atlas
GEO: Gene Expression Omnibus
TIMER: Tumor Immune Estimation Resource
CAN: copy number alteration
HCC: hepatocellular carcinoma
OS: overall survival
KEGG: Kyoto Encyclopedia of Genes and Genomes
GO: Gene Ontology
TME: tumor microenvironment
GDC: Genomic Data Commons
CPTAC: Clinical Proteomics Cancer Analysis Consortium
TMB: tumor mutational burden
MSI: microsatellite instability
GSEA: gene Set Enrichment Analysis
HPA: the Human Proteome Atlas.
