# Supplementary material for: I kappa B kinase interacting protein as a promising biomarker in pan-cancer: A multi-omics analysis
Source: Front Genet. 2023 Mar 14;14:1138137. doi: 10.3389/fgene.2023.1138137 (PMC10047260; doi:10.3389/fgene.2023.1138137)
Supplement: Supplementary file 1 [file Image1.pdf]

Supplementary materials:

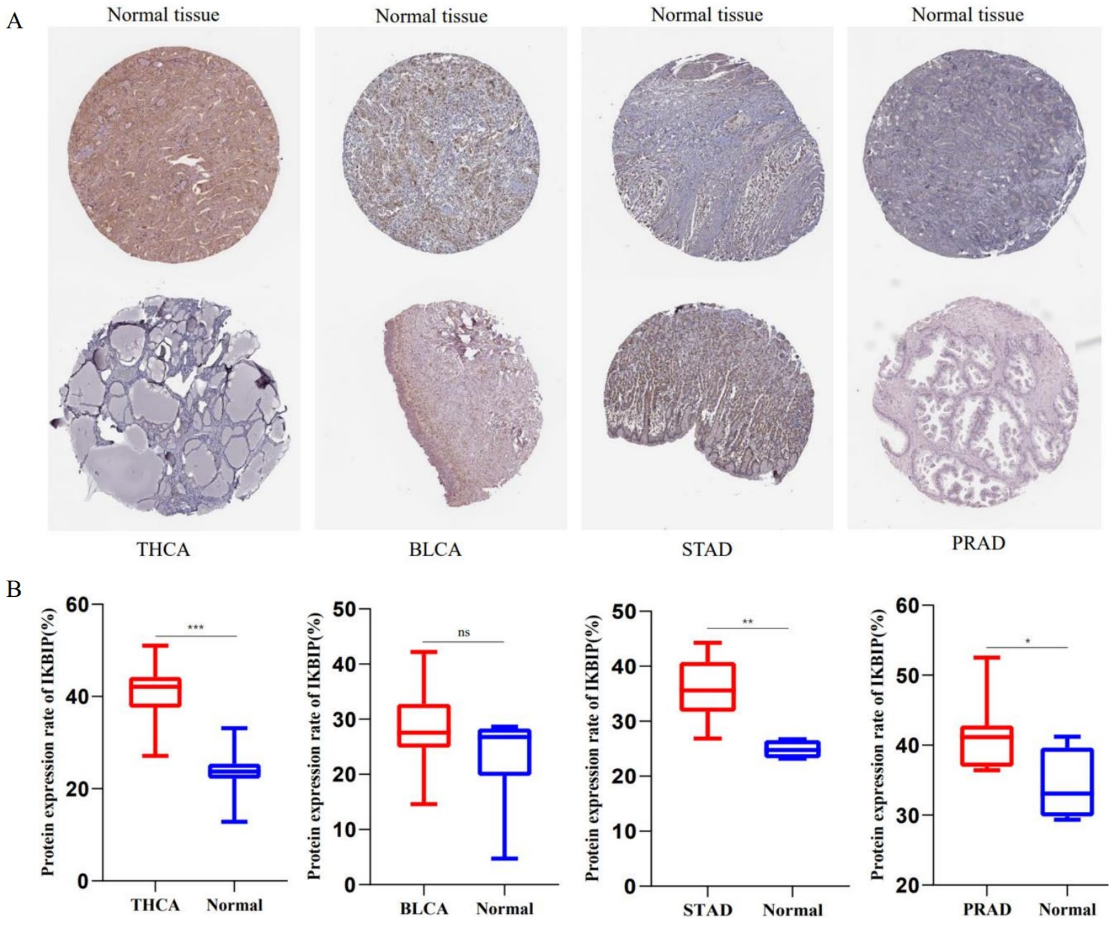

Figure S1. A Immunohistochemical pictures of the normal group (top) and tumor group show the expression of the IKBIP protein (bottom). B Quantitative analysis of IKBIP protein expression was significantly higher in THCA, STAD, and PRAD than in normal tissues, but there was no significant difference in protein expression in BLCA compared to normal tissues. (*p<0.05, **p<0.01, and ***p<0.001)

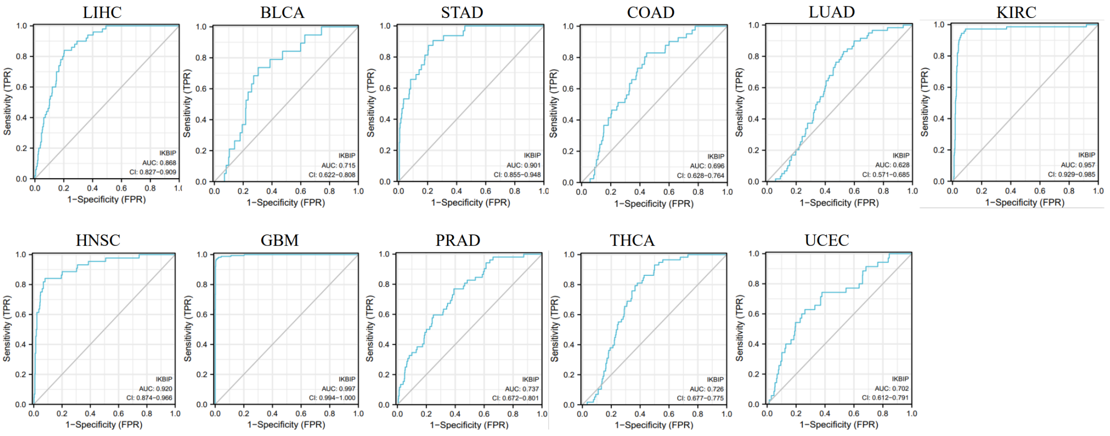

Figure S2. AUC of ROC curves verified the diagnosis performance of IKBIP in the TCGA cohort.

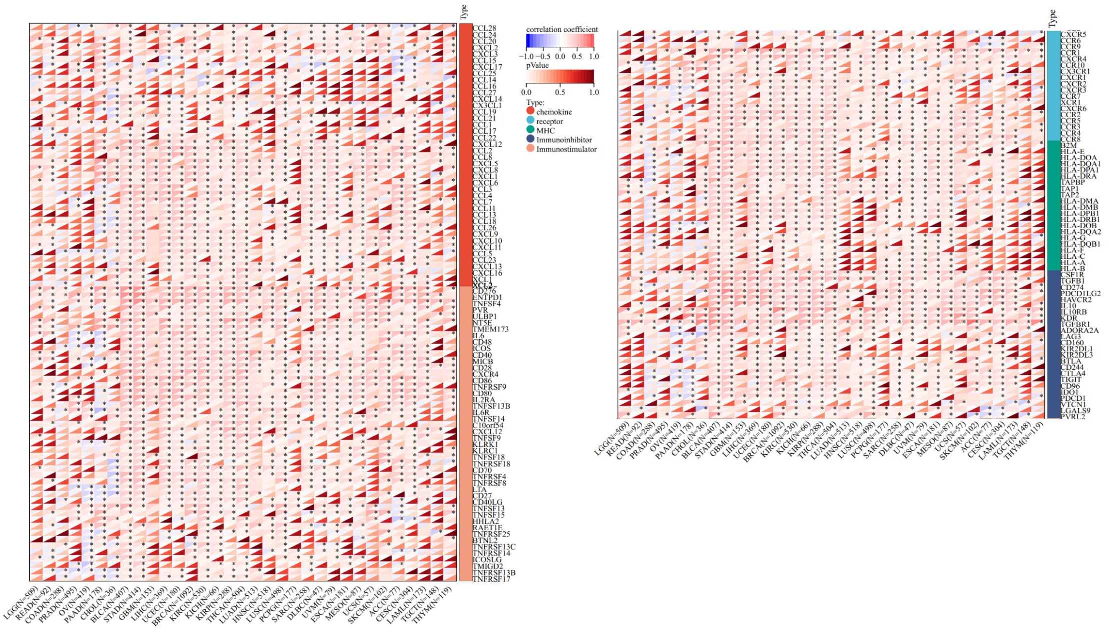

Figure S3. IKBIP is related to 150 genes that act as immunomodulators (chemokines, receptors, MHC, and immune-stimulators). (*p<0.05, **p<0.01, and ***p<0.001)

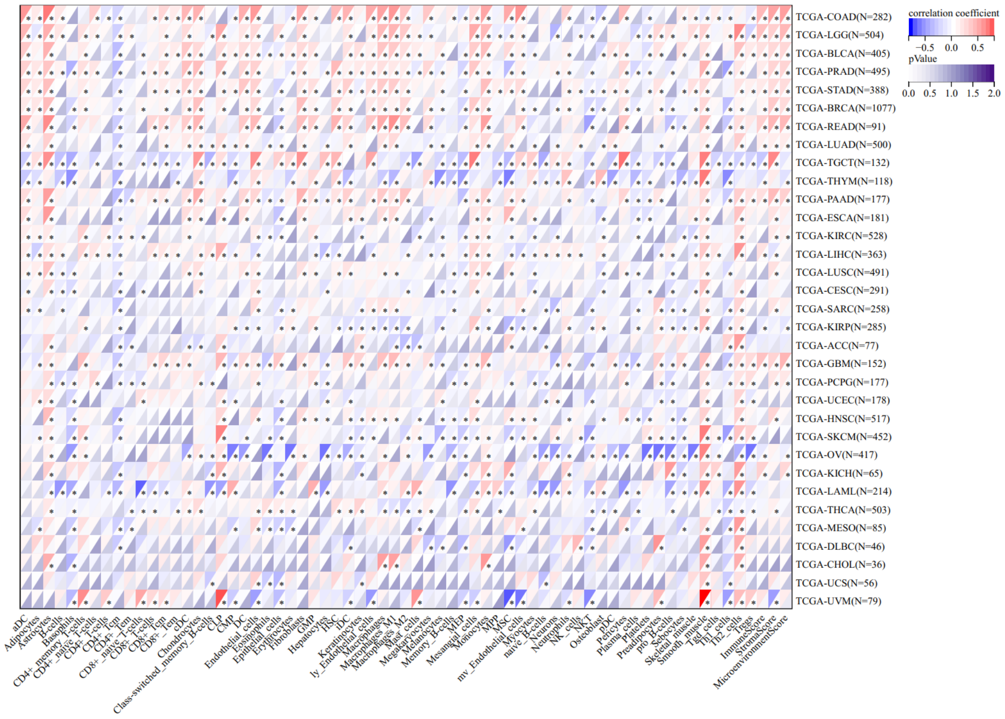

Figure S4. Results from xCell demonstrated a significant association between IKBIP expression and immune cell infiltration levels. (*p<0.05, **p<0.01, and ***p<0.001)

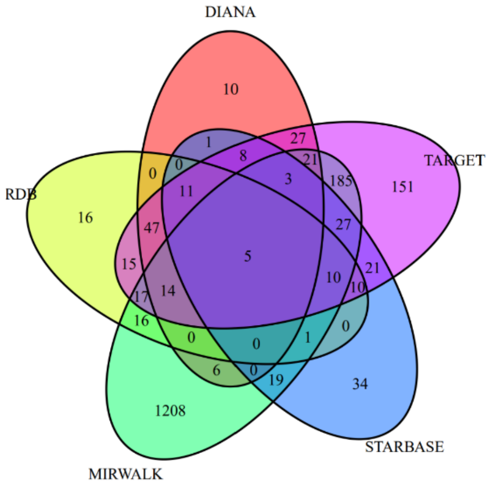

Figure S5. The upstream miRNAs of IKBIP were predicted using miRWalk, miRDB, TargetScan, DIANA-microT, and StarBase v2.0, and the intersection was obtained (nine intersection miRNAs).

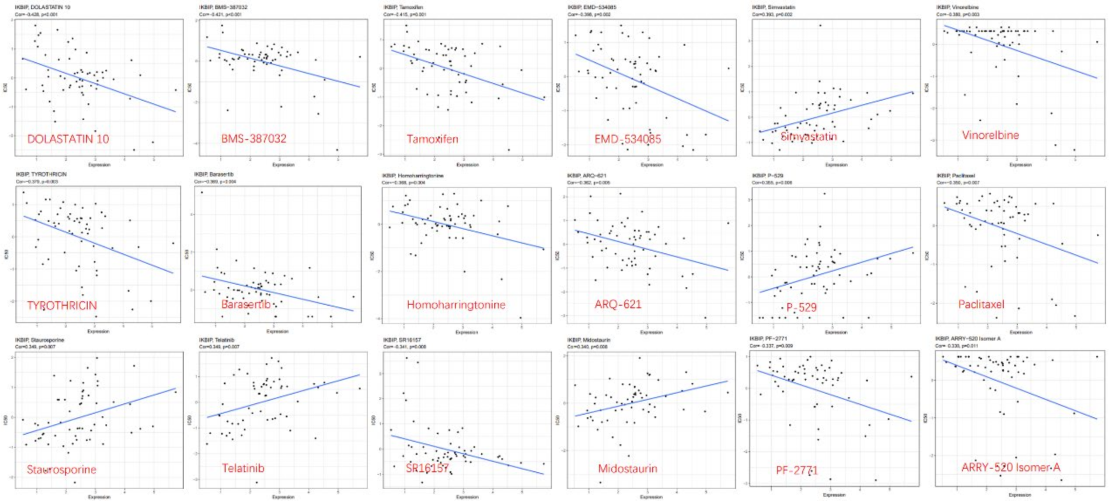

Figure S6. Drug sensitivity analysis of IKBIP. X-axis shows IKBIP expression level, Y-axis shows drug sensitivity.

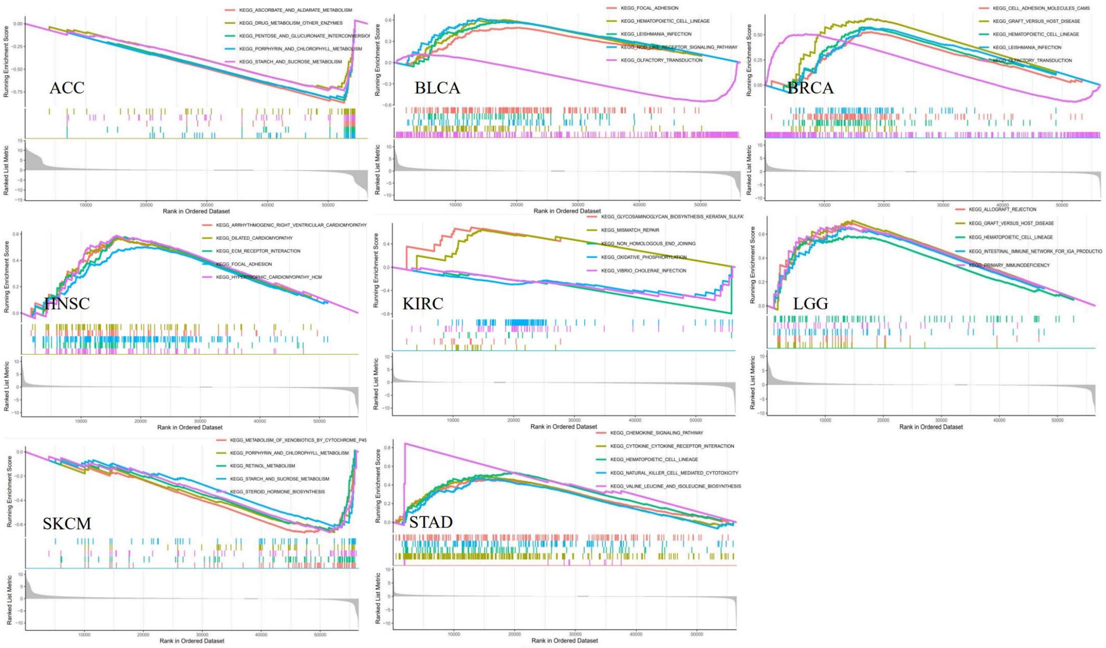

KEGG

Figure S7. IKBIP in each cancer type in the high-risk group and low-risk group was analyzed using the KEGG pathway. Different colored curves show that IKBIP regulates diverse routes or functions in various cancers. Peaks on the upward curve indicate positive regulation, while peaks on the downward curve indicate negative regulation.
